# Squeezing giant spin states via geometric phase control in cavity-assisted Raman transitions

**DOI:** 10.1038/s41598-017-12486-1

**Published:** 2017-10-09

**Authors:** Keyu Xia

**Affiliations:** 1 National Laboratory of Solid State Microstructures, College of Engineering and Applied Sciences, Nanjing University, Nanjing, 210093 China; 2 ARC Centre for Engineered Quantum Systems, Department of Physics and Astronomy, Macquarie University, NSW, 2109 Australia

## Abstract

Squeezing ensemble of spins provides a way to surpass the standard quantum limit in quantum metrology and test the fundamental physics as well, and therefore attracts broad interest. Here we propose an experimentally accessible protocol to squeeze a giant ensemble of spins via the geometric phase control (GPC). Using the cavity-assisted Raman transition (CART) in a double Λ-type system, we realize an effective Dicke model. Under the condition of vanishing effective spin transition frequency, we find a particular evolution time where the cavity decouples from the spins and the spin ensemble is squeezed considerably. Our scheme combines the CART and the GPC, and has the potential to improve the sensitivity in quantum metrology with spins by about two orders.

## Introduction

Spins, due to the merit of their long decoherence, have been widely used for ultrasensitive sensing of various signals^1–10^. However, the precision of the conventional measurement with spins is bounded by the shot noise or the SQL^11,12^. Quantum spin squeezing and entanglement can surpass the SQL and therefore boost the sensitivity in quantum measurements to approach the Heisenberg limit^11,13^.

To exploit the power of the spin-squeezed state (SSS), various methods have been proposed using quantum measurement^14–16^, quantum bath engineering^17^, converting entanglement to squeezing^18^ and cavity feedback^19,20^, typically for atomic ensembles. The state-of-the-art experiment has achieved 20 dB squeezing of half a million ultracold Rb atoms in a natural trap^14^. Recently, Bennett *et al*. show the potential to squeeze 100 nitrogen-vacancy (NV) spins in diamond via the Tavis-Cummings interaction with a nanomechanical resonator, mediated by strain^21^. Their scheme inevitably and sensitively suffers to the large thermal excitation of mechanical resonator. Zhang’s and our works show that the NV centers can also couple to a mechanical resonator mediated by a giant magnetic gradient^22,23^. This hybrid system enables to squeeze NV centers by controlling the so-called geometric phase, which is a global phase accumulated during the evolution of a quantum system. Taking the merit of the geometric phase protocol robust again various noises, the squeezing is immune to thermal excitation^22,23^. However, the giant magnetic gradient causes large Zemman splitting in NV centers and is highly localized in nanometer region. As a result, the available number of squeezed spins is limited up to 20^22,23^. Cavity-assisted Raman transition (CART) has been proposed and then demonstrated for Dicke model quantum phase transitions^24–27^. Here we aim to provide an experimentally feasible scheme to squeeze millions or even trillions of spins using CART. Our geometric-phase-based scheme has the potential to surpass the achieved squeezing degree of other schemes.

In this paper, we propose an unconventional scheme for squeezing, in a transient way, a large ensemble of spins via the geometric phase control (GPC). We couple the ensemble of ultracold alkali atoms or a superfluid gas formed in Bose-Einstein condensate (BEC) or negatively charged silicon-vacancy (SiV^−^) color centers in diamond to the cavity. Using CART, we create an effective Dicke model for the spin-photon interaction. In a special arrangement, the effective resonance frequency, *ω*_*c*_, of the cavity is much larger than the effective transition frequency of the spins. At a particular time, *t* = 2π/*ω*_*c*_, the spin and cavity decouples. At the same time, the ensemble of spins accumulates a geometric phase due to the collective interaction with the cavity and are collectively twisted along one axis of the Bloch sphere of spins. As a result, the cavity squeezes the spins considerably. Because the spins can be optically initialized to their ground state and the thermal excitation of the optical cavity is vanishing small even at room temperature, our scheme has an advantage that the thermal noise can be neglected in squeezing. Importantly, we, for the first time, combine these two powerful quantum technologies, the CART and the GPC, for achieving the challenging goal squeezing a large ensemble of spins.

This paper is organized as following: First, we present the general configuration and model for our system. After that, we discuss the special properties and the realistic parameters of three specific implementations for our numerical simulations. Then we present our numerical results. Finally, we discuss the achievable squeezing degrees of three different types of implementations and conclude our work.

## Model

**Figure 1:**
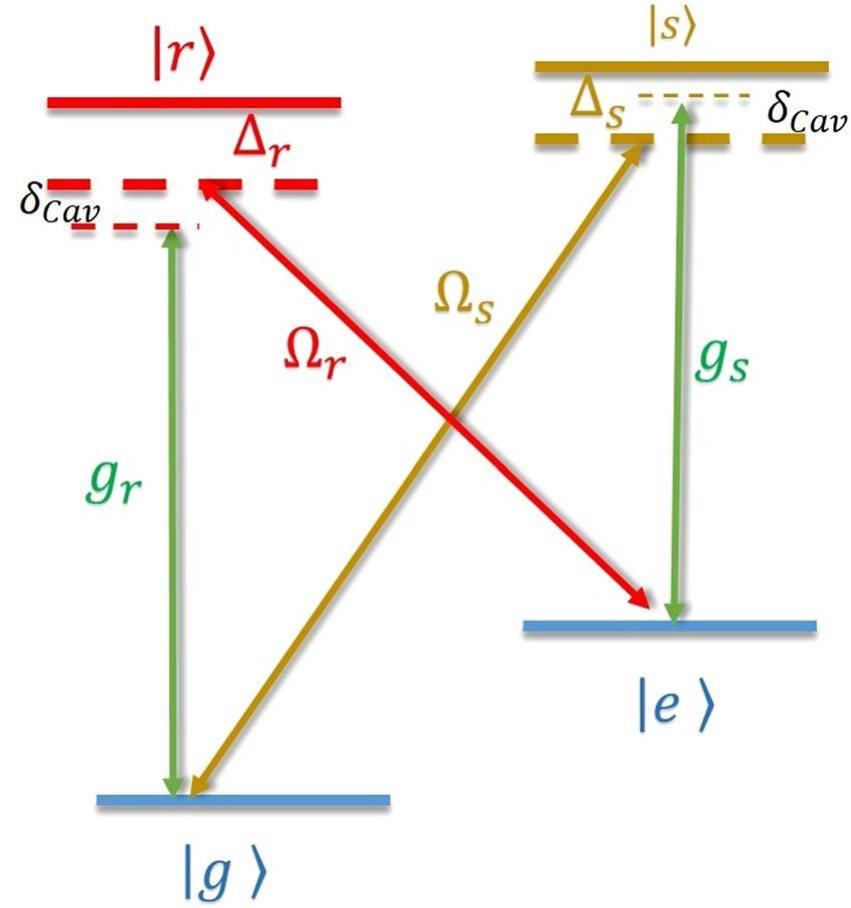
Level diagram for showing two CARTs. In combination with a cavity mode (green lines), two classical laser fields, ${{\rm{\Omega }}}_{r}$ and ${{\rm{\Omega }}}_{s}$, (red and brown) drives the spins to form CARTs between states $|e\rangle$ and $|g\rangle$.

We start the discussion of our work by describing the system. We apply the cavity electrodynamics (QED) configuration presented by Dimer *et al*.^24^, in which an ensemble of *N*_*a*_ double Λ-type systems is trapped, to form a Dicke model for our purpose of squeezing spin states. The level diagram of the system is depicted in Fig. 1. Each Λ-type system has two optical excited states $|r\rangle$ and $|s\rangle$, and two metastable states $|g\rangle$ and $|e\rangle$. The state $|j\rangle$ has energy $\hslash {\omega }_{j}$ (*j* = *r, s, g, e*). We assume that the excited states, $|r\rangle$ ($|s\rangle$) decay to the two ground states, $|g\rangle$ and $|e\rangle$, with the rates of *γ*_*rg*_ and *γ*_*re*_ (*γ*_*sg*_ and *γ*_*se*_), respectively. The cavity mode, *ĉ*, with resonance frequency *ω*_cav_ and decay rate $\kappa$, drives the transition $|g\rangle \leftrightarrow |r\rangle$ ($|e\rangle \leftrightarrow |s\rangle$) with strength ${g}_{r}$ (${g}_{s}$). The classical laser field drives the atomic transition $|e\rangle \leftrightarrow |r\rangle$ ($|g\rangle \leftrightarrow |s\rangle$) with Rabi frequency Ω_*r*_ (Ω_*s*_) and detuning ${{\rm{\Delta }}}_{r}=({\omega }_{r}-{\omega }_{e})-{\omega }_{lr}$ (${{\rm{\Delta }}}_{s}=({\omega }_{s}-{\omega }_{g})-{\omega }_{ls}$). *ω*_*lr*_ and *ω*_*ls*_ are the carrier frequencies of the laser fields Ω_*r*_ and Ω_*s*_. The paired interaction, ${g}_{r}$ and ${{\rm{\Omega }}}_{r}$, ${g}_{s}$ and ${{\rm{\Omega }}}_{s}$, forms two CARTs. Each CART drives the transition between two ground states. Combining these two CARTs, we obtain the Dicke Hamiltonian^24^ which is the key of our GPC. The original configuration has been presented for studying the quantum phase transition^24^. Here we apply it for squeezing spin states via the GPC.

We now go to derive the Dicke Hamiltonian governing the evolution of system. In the configuration depicted in Fig. 1, the transitions $|r\rangle \leftrightarrow |g\rangle$ and $|s\rangle \leftrightarrow |e\rangle$ of the *j*th atom are driven by the cavity mode with rates ${g}_{r,j}$ and ${g}_{s,j}$, respectively. The two classical laser fields, ${{\rm{\Omega }}}_{r}$ and ${{\rm{\Omega }}}_{s}$, drives the transitions $|r\rangle \leftrightarrow |e\rangle$ and $|s\rangle \leftrightarrow |g\rangle$, respectively. The Hamiltonian describing the atoms and the cavity takes the form,

1 $$\begin{array}{ll}\hat{H} & \,=\,{\omega }_{{\rm{cav}}}{\hat{c}}^{\dagger }\hat{c}+\sum _{j,l=r,s,e,g}{\omega }_{l}|{l}_{j}\rangle \langle {l}_{j}|\\  & \,\,\,\,+\sum _{j}({g}_{r,j}{e}^{-ik{r}_{j}}{\hat{c}}^{\dagger }|{g}_{j}\rangle \langle {r}_{j}|+{g}_{s,j}{e}^{-ik{r}_{j}}{\hat{c}}^{\dagger }|{e}_{j}\rangle \langle {s}_{j}|+H\mathrm{.}c\mathrm{.})\\  & \,\,\,\,+\sum _{j}(\frac{{{\rm{\Omega }}}_{r}}{2}{e}^{-i{\omega }_{lr}t}{e}^{i{k}_{lr}{r}_{j}}|{r}_{j}\rangle \langle {e}_{j}|+\frac{{{\rm{\Omega }}}_{s}}{2}{e}^{-i{\omega }_{ls}t}{e}^{i{k}_{ls}{r}_{j}}|{s}_{j}\rangle \langle {g}_{j}|+H\mathrm{.}c\mathrm{.}),\end{array}$$

where $k={\omega }_{{\rm{cav}}}/c$, ${k}_{lr}={\omega }_{lr}/c$ and ${k}_{ls}={\omega }_{ls}/c$ with $c$ the light velocity in vacuum are the wave vector of the cavity mode and the classical laser fields, ${r}_{j}$ is the position of the *j*th spin. We transform the system into the interaction picture by introducing the unitary transformation $\hat{U}(t)=\exp (-i{H}_{0}t)$ with ${H}_{0}={\sum }_{j}{\omega }_{g}|{g}_{j}\rangle \langle {g}_{j}|+{\omega }_{e}|{e}_{j}\rangle \langle {e}_{j}|$ +$({\omega }_{lr}+{\omega }_{e})|{r}_{j}\rangle \langle {r}_{j}|+({\omega }_{ls}+{\omega }_{g})|{s}_{j}\rangle \langle {s}_{j}|+{\omega }_{{\rm{cav}}}^{\text{'}}{\hat{c}}^{\dagger }\hat{c}$, as in^24^. We set ${\omega }_{ls}-{\omega }_{lr}=\mathrm{2(}{\omega }_{e}-{\omega }_{g})$ that ${\omega }_{{\rm{cav}}}^{{^{\prime} }}={\omega }_{lr}+({\omega }_{e}-{\omega }_{g})$ $=\,{\omega }_{ls}-({\omega }_{e}-{\omega }_{g})$. Thus we obtain the Hamiltonian in the interaction picture,

2 $$\begin{array}{ll}H= & {\delta }_{{\rm{cav}}}\sum _{j}({{\rm{\Delta }}}_{r}|{r}_{j}\rangle \langle {r}_{j}|+{{\rm{\Delta }}}_{s}|{s}_{j}\rangle \langle {s}_{j}|)\\  & +\sum _{j}({g}_{r,j}{e}^{-ik{r}_{j}}{\hat{c}}^{\dagger }|{g}_{j}\rangle \langle {r}_{j}|+{g}_{s,j}{e}^{-ik{r}_{j}}{\hat{c}}^{\dagger }|{e}_{j}\rangle \langle {s}_{j}|+H\mathrm{.}c\mathrm{.})\\  & +\sum _{j}(\frac{{{\rm{\Omega }}}_{r}}{2}{e}^{i{k}_{lr}{r}_{j}}|{r}_{j}\rangle \langle {e}_{j}|+\frac{{{\rm{\Omega }}}_{s}}{2}{e}^{i{k}_{ls}{r}_{j}}|{s}_{j}\rangle \langle {g}_{j}|+H\mathrm{.}c\mathrm{.}),\end{array}$$

We assume $k\approx {k}_{lr}\approx {k}_{ls}$. To a good approximation, we can assume ${g}_{r,j}={g}_{r}$ and ${g}_{s,j}={g}_{s}$ when the cavity mode is a running wave field^24^ and the waist of cavity mode is much larger than the transversal dimension of the spin sample. Taking $|{{\rm{\Delta }}}_{r,s}|\gg {{\rm{\Omega }}}_{r,s},{g}_{r,s},\gamma$, we adiabatically eliminate the optical excited states $|{r}_{j}\rangle$ and $|{s}_{j}\rangle$, and neglect the constant energy terms to arrive at the Dicke model Hamiltonian for the collective coupling of the ground states $|{g}_{j}\rangle$ and $|{e}_{j}\rangle$ ^24^,

3 $${H}_{{\rm{Dicke}}}={\omega }_{c}{\hat{c}}^{\dagger }\hat{c}+{\omega }_{q}{J}_{z}+2\sqrt{{N}_{a}}\lambda ({\hat{c}}^{\dagger }+\hat{c}){\bar{J}}_{x},$$

where ${\omega }_{c}={\delta }_{{\rm{cav}}}-\frac{1}{2}{N}_{a}(\frac{|{g}_{r}{|}^{2}}{{{\rm{\Delta }}}_{r}}+\frac{|{g}_{s}{|}^{2}}{{{\rm{\Delta }}}_{s}})$, ${\omega }_{q}=\frac{|{{\rm{\Omega }}}_{s}{|}^{2}}{4{{\rm{\Delta }}}_{s}}-\frac{|{{\rm{\Omega }}}_{r}{|}^{2}}{4{{\rm{\Delta }}}_{r}}$ caused by the ac Stark shifts. Namely, the two-photon detuning in the CARTs is ${\delta }_{{\rm{cav}}}$. Here we define the collective operators for the spins, ${J}_{z}={\sum }_{j}(|{e}_{j}\rangle \langle {e}_{j}|-|{g}_{j}\rangle \langle {g}_{j}\mathrm{|)/2}$, ${J}_{+}={J}_{-}^{\dagger }={\sum }_{j}|{e}_{j}\rangle \langle {g}_{j}|$ and ${\bar{J}}_{x}=({J}_{+}+{J}_{-}\mathrm{)/2}\sqrt{{N}_{a}}$. For our purpose of squeezing spins, we choose $\frac{|{g}_{r}{|}^{2}}{{{\rm{\Delta }}}_{r}}=\frac{|{g}_{s}{|}^{2}}{{{\rm{\Delta }}}_{s}}$ and $\lambda =\frac{{{\rm{\Omega }}}_{r}^{\ast }{g}_{r}}{2{{\rm{\Delta }}}_{s}}=\frac{{{\rm{\Omega }}}_{s}{g}_{s}^{\ast }}{2{{\rm{\Delta }}}_{s}}$ by controlling the detuning and the classical driving. Essentially, these conditions requires ${{\rm{\Delta }}}_{r}/{{\rm{\Delta }}}_{s}=|{d}_{rg}{|}^{2}/|{d}_{se}{|}^{2}$ and ${{\rm{\Omega }}}_{r}/{{\rm{\Omega }}}_{s}={d}_{rg}/{d}_{se}$ when the dipole moments ${d}_{rg,se}$, ${g}_{r,s}$ and ${{\rm{\Omega }}}_{r,s}$ are real numbers. As a results, ${\omega }_{q}\,=\,0$ is obtained. We will also investigate the case of ${\omega }_{q}\ne 0$ for a general discussion of squeezing BEC. We can consider the ensemble of spins as a resonator with annihilation operator $\hat{a}$ under the Holstein-Primakoff (HP) transformation that ${J}_{z}=({\hat{a}}^{\dagger }\hat{a}-{N}\mathrm{/2)}$, ${J}_{+}={\hat{a}}^{\dagger }\sqrt{{N}-{\hat{a}}^{\dagger }\hat{a}}$, ${J}_{-}=\sqrt{{N}-{\hat{a}}^{\dagger }\hat{a}}\hat{a}$, and ${\bar{J}}_{x}=({\hat{a}}^{\dagger }\sqrt{{\bf{I}}-{\hat{a}}^{\dagger }\hat{a}/{N}_{a}}+$ $\sqrt{{\bf{I}}-{\hat{a}}^{\dagger }\hat{a}/{N}_{a}}\hat{a}\mathrm{)/2}$ ^28,29^, where ${N}={N}_{a}{\bf{I}}$. In the ideal case of ${\omega }_{q}\,=\,0$, we rewrite the Hamiltonian in the interaction picture of ${\omega }_{c}{\hat{c}}^{\dagger }\hat{c}$ as

4 $${V}_{x}=2\sqrt{{N}_{a}}\lambda ({e}^{i{\omega }_{c}t}{\hat{c}}^{\dagger }+{e}^{-i{\omega }_{c}t}\hat{c}){\bar{J}}_{x}\mathrm{.}$$

Now we go to the GPC of the evolution of the system. By applying the Magnus’s formula^30^, the dynamics for the system is governed exactly, in the absence of decoherence, by the unitary operator ${U}_{x}(t)={e}^{i{N}_{a}\theta (t){\bar{J}}_{x}^{2}}{e}^{2\sqrt{{N}_{a}}\lambda /{\omega }_{c}(\alpha (t){\hat{c}}^{\dagger }-{\alpha }^{\ast }(t)\hat{c}){\bar{J}}_{x}}$, where $\alpha (t)=1-{e}^{i{\omega }_{c}t}$, and $\theta (t)={(\frac{2\lambda }{{\omega }_{c}})}^{2}({\omega }_{c}t-\,\sin \,{\omega }_{c}t)$. $\theta (t)$ is the accumulated geometric phase only dependent on the global geometric features of operators and is robust against random operation errors^31^. Note that the spin-cavity coupling is modulated quickly by the periodic function $\alpha (t)$. At ${t}_{m}=2m\pi /{\omega }_{c}$ for an integer $m$, $\alpha ({t}_{m})$ vanishes, $\theta ({t}_{m})=2m\pi {(\frac{2\lambda }{{\omega }_{c}})}^{2}$ and the spins decouple from the cavity. As a result, the evolution operator for the spin ensemble takes an explicit form,

5 $${U}_{x}({t}_{m})={e}^{i{N}_{a}\theta ({t}_{m}){\bar{J}}_{x}^{2}}\mathrm{.}$$

Typically $\langle {\hat{a}}^{\dagger }\hat{a}\rangle \ll {N}_{a}$, to a good approximation of ${\bar{J}}_{x}\approx ({\hat{a}}^{\dagger }+\hat{a}\mathrm{)/2}$, so we can treat the operator ${U}_{x}({t}_{m})$ as a squeezing operator with a degree of squeezing of ${N}_{a}\theta ({t}_{m}\mathrm{)/2}$ by negecting the phase rotation due to ${\hat{a}}^{\dagger }\hat{a}+\hat{a}{\hat{a}}^{\dagger }$. Given the initial state $|{\rm{\Psi }}\mathrm{(0)}\rangle$ for the spin ensemble, the generated state after one period, i.e. at ${t}_{1}$ is $|{\rm{\Psi }}({t}_{1})\rangle ={U}_{x}({t}_{1})|{\rm{\Psi }}\mathrm{(0)}\rangle$. It is noticeable that the squeezing degree of the SSS only depends on the accumulated geometric phase $\theta ({t}_{1})$, which can be adjusted with the classical driving and the detuning.

The power of our protocol in squeezing spins is limited by the discrepancy of ${\omega }_{q}$ from zero and the decoherence of system. Although we set ${\omega }_{q}=0$ for the analysis of ideal GPC, the protocol actually works efficiently when ${\omega }_{c}\gg {\omega }_{q}$. In comparison with the protocol using a mechanical resonator to enable the GPC^22,23^, the crucially detrimental thermal noise is negligible in our scheme because the thermal excitation of the optical cavity is vanishing small and the spins can be optically polarized in the ground state $|{g}_{j}\rangle$. The decay of excited states $|{r}_{j}\rangle$ and $|{s}_{j}\rangle$ can introduce some decoherence to the evolution via CARTs but is suppressed by the large detuning^32^. Threfore, the decay of the cavity is the main decoherence source. Another decoherence source is the pure dephasing, ${{\rm{\Gamma }}}_{\varphi }$, of the ground state $|{e}_{j}\rangle$. To taking into account the influence of the imperfection in ${\omega }_{q}$ and the decoherence, we numerically solve the quantum Langevin equation in the HP picture^32^ (also see the appendix),

6 $$\partial \rho /\partial t=-i[{H}_{{\rm{Dicke}}},\rho ]+{ {\mathcal L} }(\sqrt{{{\rm{\Gamma }}}_{\varphi }\mathrm{/2}}{J}_{z})\rho +{{ {\mathcal L} }}_{c}(\sqrt{\kappa }\hat{c})\rho ,$$

where $${{ {\mathcal L} }}_{c}(\hat{A})\rho =\hat{A}\rho {\hat{A}}^{\dagger }-\frac{1}{2}{\hat{A}}^{\dagger }\hat{A}\rho -\frac{1}{2}\rho {\hat{A}}^{\dagger }\hat{A}.$$

In our systems investigated below, the dark states of spins are rarely excited, thanks to the small inhomogeneous broadening of the excited state. Therefore, we focus on the symmetric states with the total spin $J={N}_{a}\mathrm{/2}$. The state of spin ensemble can be fully described by set of the Dicke state $|J,m\rangle$ with $m\in \{-J,-J+\mathrm{1,}\cdots ,J-\mathrm{1,}\,J\}$ in the spin picture, which is equivalent to the Fock state $|J+m\rangle$ in the Bosonic or HP picture. In the later, the squeezing degree of spin states $\{|g\rangle ,|e\rangle \}$ of spin ensemble can be evaluated by the squeezing parameter defined by Wineland *et al*. as ${\xi }_{R}^{2}={(\frac{{N}_{a}}{\mathrm{2|}\langle \overrightarrow{J}\rangle |})}^{2}{\xi }_{s}^{2}\,$ ^13^, where $|\langle \overrightarrow{J}\rangle |=\sqrt{{\langle {J}_{x}\rangle }^{2}+{\langle {J}_{y}\rangle }^{2}+{\langle {J}_{z}\rangle }^{2}}$ and the squeezing parameter ${\xi }_{s}^{2}=1+2\langle {\hat{a}}^{\dagger }\hat{a}\rangle -2\frac{\langle {({\hat{a}}^{\dagger }\hat{a})}^{2}\rangle }{{N}_{a}}-\mathrm{2|}\langle {\bar{J}}_{x}^{2}\rangle |$ is given by Kitagawa and Ueda^13^. The squeezing is optimal at ${\theta }_{{\rm{opt}}}={6}^{-\mathrm{1/6}}{(N\mathrm{/2)}}^{-\mathrm{2/3}}\,$ ^23^. Correspondingly, the phase uncertainty in quantum metrology with such SSS can be reduced down to $\delta \varphi ={\xi }_{R}/\sqrt{N}$, improved by a factor of ${\xi }_{R}$.

## Parameters for implementations

Here lets first briefly discuss three possible implementations using ultracold alkali atoms, negatively charged Silicon-vacancy (SiV^−^) centers in diamond or a superfluid gas formed in Bose-Einstein condensate (BEC). All three systems for implementations can be effectively treated as an ensemble of spin-$\mathrm{1/2}$ systems in the Dicke model. As an example, we consider an ensemble of ultracold ^87^Rb atoms for the first implementation^25,33^. We choose $|r\rangle ={\mathrm{|5}}^{2}{P}_{\mathrm{3/2}},{F}^{\text{'}}=\mathrm{2,}\,{m}_{{F}^{\text{'}}}=1\rangle$, $|s\rangle ={\mathrm{|5}}^{2}{P}_{\mathrm{3/2}},{F}^{\text{'}}=\mathrm{2,}\,{m}_{{F}^{\text{'}}}=2\rangle$, $|g\rangle ={\mathrm{|5}}^{2}{S}_{\mathrm{1/2}},F=\mathrm{1,}\,{m}_{F}=1\rangle$ and $|e\rangle ={\mathrm{|5}}^{2}{S}_{\mathrm{1/2}},F=\mathrm{2,}\,{m}_{F}=2\rangle$ in the ${D}_{2}$ line of ^87^Rb atom. According to atomic data^33^, the dipole moments are ${d}_{rg}={d}_{re}=-\sqrt{\mathrm{1/8}}d$ for the transitions $|r\rangle \leftrightarrow |g\rangle$ and $|r\rangle \leftrightarrow |e\rangle$, ${d}_{sg}=\sqrt{\mathrm{1/4}}d$ for $|s\rangle \leftrightarrow |g\rangle$, and ${d}_{se}=\sqrt{\mathrm{1/6}}d$ for $|s\rangle \leftrightarrow |e\rangle$, with $d=3.584\times {10}^{-29}\,{\rm{C}}\cdot {\rm{m}}$. In such configuration, the cavity mode can be a linear-polarized field and the cavity-atom interaction is strong due to the large dipole-dipole moments. Other hyperfine levels can be effectively decoupled due to the large detuning which can also be adjusted with a constant magnetic field ${B}_{c}$ ^25^. The each excited state decays at a rate of $\gamma \sim 2\pi \times \mathrm{6\ }\,{\rm{MHz}}$ ^25,33^, yielding ${\gamma }_{rg}={\gamma }_{re}=2\pi \times \mathrm{3\ }\,{\rm{MHz}}$, ${\gamma }_{rg}=2\pi \times 3.6\,{\rm{MHz}}$, and ${\gamma }_{se}=2\pi \times 2.4\,{\rm{MHz}}$ for different branches. Interestingly, we can also squeeze an ensemble of SiV^−^ centers in diamond trapped in a cavity^6^. The SiV^−^ centers in diamond cut with $\mathrm{\{111\}}$ surface have shown a double ${\rm{\Lambda }}$-type configuration^34–36^. To use SiV centers for our scheme, we take $|s\rangle =|{}^{2}{\bf{E}}_{u},{e}_{-}^{u},\uparrow \rangle$, $|r\rangle =|{}^{2}{\bf{E}}_{u},{e}_{-}^{u},\downarrow \rangle$, $|e\rangle =|{}^{2}{\bf{E}}_{g},{e}_{+}^{u},\uparrow \rangle$, $|g\rangle =|{}^{2}{\bf{E}}_{g},{e}_{+}^{u},\downarrow \rangle$, respectively^37^. The relaxation rate, ${\rm{\Gamma }}$, of the spin ground state is negligible ($2.4m{s}^{-1}$), but the pure dephasing, ${{\rm{\Gamma }}}_{\varphi }$, is about $2\pi \times 3.5\,{\rm{MHz}}$ ^34,35^. While, the relaxation of the optical excited states, $|r\rangle$ and $|s\rangle$, is negligible at cryogenic temperature^37^. We assume ${d}_{rg}={d}_{re}={d}_{sg}={d}_{se}$. At $T\,=\,\mathrm{1\ }$, we can take ${\gamma }_{rg}={\gamma }_{re}={\gamma }_{sg}={\gamma }_{se}=2\pi \times 3.7\,{\rm{MHz}}$. More remarkably, our protocol can squeeze the momentum of a superfluid gas which can also construct the double $\Lambda$-type configuration^26^, taking $|r\rangle =|\pm \hslash k,\,0\rangle ^{\prime}$, $|s\rangle =\mathrm{|0,}\pm \hslash k\rangle ^{\prime}$, $|g\rangle =\mathrm{|0,}\,0\rangle$ and $|e\rangle =|\pm \hslash k,\pm \hslash k\rangle$. The Dicke model driving the effective transition between $\mathrm{|0,}\,0\rangle$, the atomic zero-momentum state, and $|\pm \hslash k,\pm \hslash k\rangle$, the symmetric superposition of momentum states can be created via the CART. In this, we can squeeze the macroscopic momentum of a BEC in a self-organized supersolid phase, which can only be realized in an ensemble of atoms cooled into a BEC. The effective energies of the cavity and the spin are controlled via the optical trapping potential, the photon-spin coupling, the detuning ${{\rm{\Delta }}}_{c}$ and the atom-induced dispersive shift of the cavity resonance $UB$ ^26^. The energy of the state $|\pm \hslash k,\pm \hslash k\rangle$ is lifted relative to the state $\mathrm{|0,}\,0\rangle$ by twice the recoil energy that ${\omega }_{q}=2\pi \times 28.6\,{\rm{kHz}}$ ^26^. While the effective energy, $\hslash {\omega }_{c}=\hslash {{\rm{\Delta }}}_{c}-UB$ is typically much larger than $\hslash {\omega }_{q}$. In the experiment, the single-atom coupling $\eta  > 2\pi \times 0.9$ kHz is achieved.

## Results

**Figure 2:**
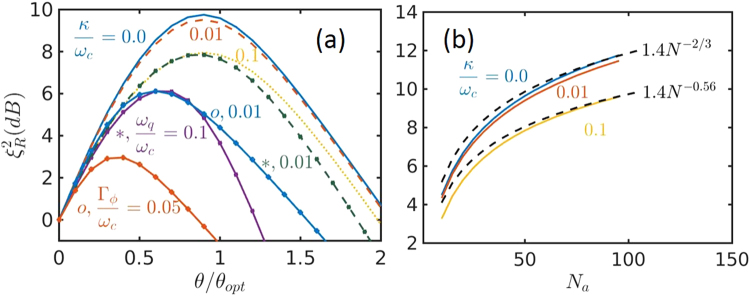
(**a**) Squeezing parameter ${\xi }_{R}^{2}$ for $N=50$ spins as a function of the geometric phase, $\theta$, at different cavity decay rate, $\kappa$ (lines without markers, ${\omega }_{q}={{\rm{\Gamma }}}_{\phi }=0$), spin transition frequency, ${\omega }_{q}$ (grouped lines with $\kappa ={{\rm{\Gamma }}}_{\varphi }=0$), the pure dephasing, ${{\rm{\Gamma }}}_{\varphi }$ (grouped lines with o markers, ${\omega }_{q}=\kappa =0$); (**b**) Squeezing parameter ${\xi }_{R}^{2}$ as a function of ${N}_{a}$ at different $\kappa$. ${{\rm{\Gamma }}}_{\varphi }=\mathrm{0,}\,{\omega }_{q}=0$ in (**b**).

Next we go to evaluate the squeezing parameter by solving the master equation Eq. (6). The cavity decay and the imperfection in ${\omega }_{q}$ dominantly limit the attainable squeezing parameter. We first study the squeezing parameter for ${N}_{a}=50$ spins at time ${t}_{1}=2\pi /{\omega }_{c}$ for different ratios $\kappa /{\omega }_{c}$ and ${\omega }_{q}/{\omega }_{c}$, as shown in Fig. 2(a). The squeezing is maximal around ${\theta }_{{\rm{opt}}}$ in the case of small ${\omega }_{q}/{\omega }_{c}$. When $\kappa =0$ and ${\omega }_{q}=0$, we obtain ${\xi }_{R}^{2}=\mathrm{9.6\ }$dB. The squeezing parameter reduces slightly for $\kappa \le 0.01\,{\omega }_{c}$ (${\omega }_{q}=0$). Even when $\kappa =0.1\,{\omega }_{c}$, ${\xi }_{R}^{2}=\mathrm{7.7\ }$dB is still achieved. In contrast, the imperfection in ${\omega }_{q}$ has stronger effect on the squeezing. The squeezing parameter for $\kappa =0$ and ${\omega }_{q}/{\omega }_{c}=0.01$ is very close to that for $\kappa /{\omega }_{c}=0.1$ and ${\omega }_{q}=0$, while it is deteriorated considerably when ${\omega }_{q}$ increases to $0.1\,{\omega }_{c}$. In this case, the maximal available squeezing parameter decreases to ${\xi }_{R}^{2}=\mathrm{6.1\ }$dB at a reduced optimal geometric phase of $\theta =0.7\,{\theta }_{{\rm{opt}}}$. In experiments, we can adjust the classical driving and the detuning so that ${\omega }_{q} < 0.01\,{\omega }_{c}$ to guarantee the optimal squeezing at $\theta \approx {\theta }_{{\rm{opt}}}$. The pure dephasing has the strongest influence on squeezing because it destroys the coherence among spins. A small pure dephasing of ${{\rm{\Gamma }}}_{\varphi }/{\omega }_{c}=0.01$ causes the maximal squeezing parameter to decrease from ~10 dB at $\theta ={\theta }_{{\rm{opt}}}$ to $6.1$ dB at $\theta =0.6{\theta }_{{\rm{opt}}}$. When ${{\rm{\Gamma }}}_{\phi }/{\omega }_{c}=0.05$, the maximal squeezing degree reduces by $\mathrm{50 \% }$, to $3$ dB.

It is always desired to provide a prediction for the attainable squeezing parameter for a large ensemble. To provide such prediction, we calculate the squeezing parameter as the number of spins increasing, see Fig. 2(b). Considering ${\omega }_{q}/{\omega }_{c}\ll 1$ available in most cases, we set ${\omega }_{q}=0$ for simplicity. The squeezing parameter is well fitted by ${\xi }_{R}^{2}=1.4{N}^{-\mathrm{2/3}}$ when $\kappa /{\omega }_{c}\le 0.01$. It decreases to $1.4{N}^{-0.56}$ with increasing the cavity decay to $\kappa /{\omega }_{c}=0.1$. Typically, $\kappa /{\omega }_{c}\le 0.01$ is achievable using current available experimental technology for ${N}_{a}\sim {10}^{6}$ ultracold atoms. It means that our GPC protocol can achieve a phase uncertainty $\delta \varphi \propto {N}^{-\mathrm{5/6}}$, approaching the Heisenberg limit of $\delta \varphi \propto {N}^{-1}$.

In above investigation, we neglect the small decoherence terms of spins. Next, we investigate the available squeezing degree for up to $100$ spins by solving the master equation with the spin decoherence and using experimental available numbers for parameters. In doing so, we can provide a rough estimation of the achievable squeezing parameter for ${10}^{6}$ spins by fitting the numerical data. We first find the geometric phase ${\theta }_{{\rm{\max }}}$ to achieve the maximal squeezing degree for ${N}_{a}=50$ spins. It is found that ${\theta }_{{\rm{\max }}}={\theta }_{{\rm{opt}}}$ for cold Rb atoms, ${\theta }_{{\rm{\max }}}=0.8{\theta }_{{\rm{opt}}}$ for BEC and ${\theta }_{{\rm{\max }}}=0.5{\theta }_{{\rm{opt}}}$ for SiV^−^ centers. Then we calculate the squeezing parameter as ${N}_{a}$ varying but with $\theta ={\theta }_{{\rm{\max }}}$ fixed. In all of three implementations, we set ${{\rm{\Omega }}}_{r}^{2}/{{\rm{\Delta }}}_{r}^{2}={{\rm{\Omega }}}_{s}^{2}/{{\rm{\Delta }}}_{s}^{2}\, < \,0.001$ for simplicity.

**Figure 3:**
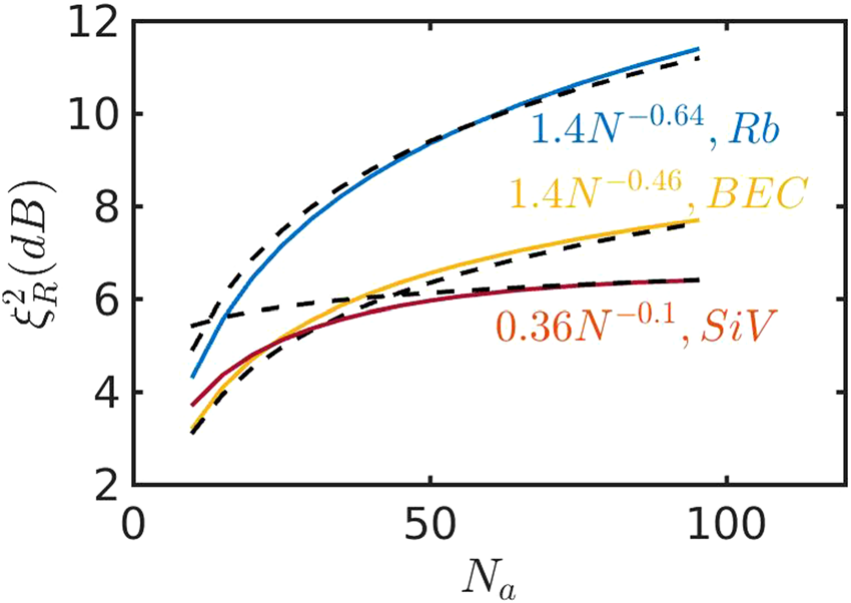
Squeezing parameter at ${\theta }_{{\rm{\max }}}$ as a function of ${N}_{a}$ for three implementations using Rb atoms (blue line), BEC (yellow line), and SiV centers (Fuchsia). ${\omega }_{c}=2\pi \times \mathrm{5.88\ }$ MHz, $\kappa =2\pi \times 70$ kHz, ${\omega }_{q}=0$, ${\theta }_{{\rm{\max }}}={\theta }_{{\rm{opt}}}$ for Rb atoms, ${\omega }_{c}=2\pi \times 500$ kHz, $\kappa =2\pi \times 70$ kHz, ${\omega }_{q}=2\pi \times 28.6$ kHz, ${\theta }_{{\rm{\max }}}=0.8{\theta }_{{\rm{opt}}}$ for BEC and ${\omega }_{c}=2\pi \times 350$ MHz, $\kappa =2\pi \times 1$ MHz, ${\omega }_{q}=0$, ${\theta }_{{\rm{\max }}}=0.5{\theta }_{{\rm{opt}}}$ for SiV centers. The lines are fitted (black dashed lines) with ${\xi }_{R}^{2}=1.4{N}^{-0.64}$ for Rb atoms, ${\xi }_{R}^{2}=1.4{N}^{-0.46}$ for BEC and ${\xi }_{R}^{2}=0.36{N}^{-0.1}$ for SiV^−^ centers.

As seen from Fig. 3, the largest squeezing of ${\xi }_{R}^{2}=1.4{N}^{-0.64}$ can be expected using an ensemble of cold alkali atoms, because the total decoherence of ground states of atoms is small and the effective transition frequency ${\omega }_{q}$ can be vanishing small. Due to the large pure dephasing of SiV centers, we can only achieve squeezing of $0.36{N}^{-0.1}$. According to^26^, the decoherence of BEC is negligible but ${\omega }_{q}=2{\omega }_{r}$ is nonzero. Taking ${\omega }_{q}=2\pi \times 28.6$ kHz^26^, we obtain the squeezing parameter of ${\xi }_{R}^{2}=1.4{N}^{-0.46}$.

## Discussion and Conclusion

Our spin-squeezing protocol via the GPC can be realized in various systems. For example, we can squeeze ${N}_{a}={10}^{6}$ cold Rb atoms. Using the experimentally available parameters^14,25^, we choose ${\omega }_{c}=2\pi \times 5.88$ MHz, $\kappa =2\pi \times 70$ kHz, ${\omega }_{q}=0$, ${g}_{r}=-\sqrt{\mathrm{3/4}}{g}_{s}=2\pi \times 1.1$ MHz, ${{\rm{\Delta }}}_{s}=\frac{4}{3}{{\rm{\Delta }}}_{r}=2\pi \times 5$ GHz, ${{\rm{\Omega }}}_{s}=\frac{{{\rm{\Delta }}}_{s}}{50}$ and ${{\rm{\Omega }}}_{r}=-\sqrt{\frac{3}{4}{{\rm{\Omega }}}_{s}}$ yielding $\lambda \mathrm{/2}\pi =-12.7$ kHz, and $\frac{|{g}_{s}|}{{{\rm{\Delta }}}_{s}},\frac{|{g}_{r}|}{{{\rm{\Delta }}}_{r}} < 3\times {10}^{-4}$, $\frac{{{\rm{\Omega }}}_{r}}{2{{\rm{\Delta }}}_{r}}\sim -1.1\times {10}^{-2},\frac{{{\rm{\Omega }}}_{s}}{2{{\rm{\Delta }}}_{s}}\sim -8.7\times {10}^{-3}$. According to the prediction in Fig. 3, the ensemble of atoms can be squeezed by ${\xi }_{R}^{2}\approx 37$dB, and the phase uncertainty in measurement with squeezed spins is $\delta \varphi \sim \mathrm{1/}{N}^{-0.82}$, close to the Heisenberg limit. If we trap billion^38^ cold atoms in the cavity, we are potentially able to obtain a squeeze degree of ${\xi }_{R}^{2}=56$ dB. The superfluid gas has the smallest decoherence but nonzero ${\omega }_{q}=2\pi \times 28.6$ kHz^26^. We take, $\kappa =2\pi \times 70$ kHz, ${{\rm{\Delta }}}_{c}\mathrm{/2}\pi =-4$ MHz, $UB\mathrm{/2}\pi =-3.5$ yielding ${\omega }_{c}\mathrm{/2}\pi =500$ kHz, and assume $\lambda =2\pi \times 0.88$ kHz. Correspondingly, the superfluid gas including ${10}^{6}$ ultracold atoms can be squeezed by ${\xi }_{R}^{2}\approx 26$ dB. It is worth noting that this is the first proposal for quantum squeezing momentum of BEC. Our protocol can only squeeze one-million SiV^−^ centers by $10.4$ because SiV^−^ centers has a pure dephasing of ${{\rm{\Gamma }}}_{\varphi }\mathrm{/2}\pi =3.5$ MHz^34,35^. To achieve it, we take $\kappa =2\pi \times 1$ MHz, ${\omega }_{c}=2\pi \times 350$ MHz, ${\rm{\Delta }}={{\rm{\Delta }}}_{r}={{\rm{\Delta }}}_{s}=2\pi \times 10$ GHz, ${{\rm{\Omega }}}_{r}={{\rm{\Omega }}}_{s}={\rm{\Delta }}\mathrm{/30}$, and a large single-atom coupling ${g}_{r}={g}_{s}=2\pi \times 46$ MHz, leading to $\frac{|{g}_{s}|}{{{\rm{\Delta }}}_{s}}=\frac{|{g}_{r}|}{{{\rm{\Delta }}}_{r}}=4.6\times {10}^{-3}$, $\frac{{{\rm{\Omega }}}_{r}}{2{{\rm{\Delta }}}_{r}}=\frac{{{\rm{\Omega }}}_{s}}{2{{\rm{\Delta }}}_{s}}=0.017$.

Using the CARTs in spins, we have proposed a GPC scheme to efficiently squeeze ensemble of spin. The available squeezing degree with increasing the number of spins has been numerically studied. Our scheme has the potential to surpass the experimental record up to date. The protocol is free of the detrimental thermal noise which heavily destroys the squeezing in mechanical resonator-based schemes. Our scheme paves a way to prepare the quantum state of a large ensemble of spins for achieving ultrasensitive quantum sensing.

It is worth nothing that the inaccuracy in the timing control may lead to a tiny nonzero $\alpha ({t}_{m})$ and subsequently reduces the degree of squeezing. However, this effect of error in timing control is small, in particular, for small ${\omega }_{c}$ in our configuration. Moreover, this “one-time” error happens only when the operation is switched off but won’t accumulate during the whole geometric phase control.

## Electronic supplementary material

Squeezing giant spin states via geometric phase control in cavity-assisted Raman transitions
