# Supplementary material for: Squeezing giant spin states via geometric phase control in cavity-assisted Raman transitions
Source: Sci Rep. 2017 Oct 9;7:12836. doi: 10.1038/s41598-017-12486-1 (PMC5634490; doi:10.1038/s41598-017-12486-1)
Supplement: Supplementary file 1 — Squeezing giant spin states via geometric phase control in cavity-assisted Raman transitions [file 41598_2017_12486_MOESM1_ESM.pdf]

Supplementary Materials

Squeezing giant spin states via geometric phase control in cavity-assisted Raman transitions

Keyu Xia[1,2,*]

[1]*College of Engineering and Applied Sciences,*
Nanjing University, Nanjing 210008, China

[2]*ARC Centre for Engineered Quantum Systems,*
Department of Physics and Astronomy,
Macquarie University, NSW 2109, Australia

In this supplementary material, we will derive the Dicke Hamiltonian H_{Dicke} for the collective coupling between the ensemble of spins and the cavity, and the decoherence described by Lindblad superoperators in the HP picture.

The motion of the atoms and the cavity in our configuration can be described by the Hamiltonian taking the form,

$$\begin{aligned}\hat{H} = \delta_{\text{cav}} \sum_j (\Delta_r |r_j\rangle\langle r_j| + \Delta_s |s_j\rangle\langle s_j|) \\ + \sum_j \left(g_{r,j} e^{-ikr_j} \hat{c}^\dagger |g_j\rangle\langle r_j| + g_{s,j} e^{-ikr_j} \hat{c}^\dagger |e_j\rangle\langle s_j| + H.c. \right) \\ + \sum_j \left(\frac{\Omega_r}{2} e^{ik_{lr} r_j} |r_j\rangle\langle e_j| + \frac{\Omega_s}{2} e^{ik_{ls} r_j} |s_j\rangle\langle g_j| + H.c. \right) ,\end{aligned} \tag{1}$$

with $\Delta_r = (\omega_r - \omega_e) - \omega_{lr}$, $\Delta_s = (\omega_s - \omega_g) - \omega_{ls}$ and $\delta_{\text{cav}} = \omega_{\text{cav}} - (\omega_{lr} + \omega_{ls})/2$. The two-photon detuning in the CART is then given by δ_{cav}.

The dynamics of the atom-cavity system is described by a master equation of Lindblad form

$$\dot{\rho} = -i[\hat{H}, \rho] + \mathscr{L}(\sqrt{\kappa} \hat{c})\rho + \mathscr{L}_\phi \rho + \sum_{k,j} \mathscr{L}(\hat{L}_{k,j})\rho , \tag{2}$$

where $\mathscr{L}(\hat{A})\rho = \hat{A}\rho\hat{A}^\dagger - \frac{1}{2}(\hat{A}^\dagger\hat{A}\rho + \rho\hat{A}^\dagger\hat{A})$ and $\hat{L}_{k,j} \in \{ \sqrt{\gamma_{rg}}|g_j\rangle\langle r_j|, \sqrt{\gamma_{re}}|e_j\rangle\langle r_j|, \sqrt{\gamma_{sg}}|g_j\rangle\langle s_j|, \sqrt{\gamma_{se}}|e_j\rangle\langle s_j| \}$ describes the decoherence of the cavity and the jth atom. $\mathscr{L}(\sqrt{\kappa}\hat{c})\rho$ is for taking into account the decay of the cavity, and $\mathscr{L}_\phi \rho$ for the pure dephasing of the spins.

Hamiltonian for CARTs

Now we go to derive the Hamiltonian for the CART describing the interaction of the cavity and effective two-level spins. To do so, we use the project-operator method of Feshbach [1] to structure the Hilbert space of atoms into two subspaces, one for the ground states and one for the excited states, represented by the projection operators P_g and P_e, with $P_g + P_e = \mathbf{I}$ and $P_g P_e = 0$. Accordingly, we can divide the Hamiltonian Eq. (1) into four parts:

$$\hat{H}_p = \hat{H}_g + \hat{H}_e + \hat{V}_+ + \hat{V}_- , \tag{3}$$

where, $\hat{H}_g = P_g \hat{H} P_g$ labels the interactions inside the ground state subspace, and $\hat{H}_e = P_e \hat{H} P_e$ for the interactions inside the excited state subspace. These two subspaces are connected by the

perturbative excitations $\hat{V}_+ = P_e \hat{H} P_g$ and the deexcitations $\hat{V}_- = P_g \hat{H} P_e$ ($\hat{V}_+^\dagger = \hat{V}_-$). We have

$$\begin{aligned}
\hat{H}_e &= \sum_j (\Delta_r |r_j\rangle\langle r_j| + \Delta_s |s_j\rangle\langle s_j|) \\
\hat{H}_g &= 0 \\
\hat{V}_{+,j}^{(e)} &= g_{s,j} e^{ikr_j} \hat{c} |s_j\rangle\langle e_j| + \frac{\Omega_r}{2} e^{ik_{lr} r_j} |r_j\rangle\langle e_j| \\
\hat{V}_{+,j}^{(g)} &= g_{r,j} e^{ikr_j} \hat{c} |r_j\rangle\langle g_j| + \frac{\Omega_s}{2} e^{ik_{ls} r_j} |s_j\rangle\langle g_j| \ ,
\end{aligned} \tag{4}$$

and $\hat{V}_+ = \sum_j \hat{V}_{+,j}^{(e)} + \hat{V}_{+,j}^{(g)}$.

We adiabatically eliminate the excited states $|r\rangle$ and $|s\rangle$ in the dispersive coupling regime. Thus in the picture only involving the ground-state subspace, the master question is reduced to the form [1]

$$\dot{\rho} = -i[\delta_{\text{cav}} \hat{c}^\dagger \hat{c} + \hat{H}_{\text{eff}}, \rho] + \mathscr{L}(\sqrt{\kappa} \hat{c})\rho + \mathscr{L}_\phi \rho + \sum_{k,j} \mathscr{L}(\hat{L}_{(\text{eff})}^{(k,j)})\rho \ . \tag{5}$$

$\hat{H}_{\text{eff}}$ and $\hat{L}_{\text{eff}}^{(k,j)}$ are the effective Hamiltonian and Lindblad operators to be derived below.

Dicke Hamiltonian

The effective Hamiltonian in Eq. (5) is given by [1]

$$\hat{H}_{\text{eff}} = -\frac{1}{2}\left[\hat{V}_- \sum_{l=g,e} \left(\hat{H}_{\text{NH}}^{(l)}\right)^{-1} \hat{V}_+^{(l)} + H.c.\right] + \hat{H}_g \ . \tag{6}$$

$\hat{H}_{\text{NH}}^{(g)} = \hat{H}_{\text{NH}}^{(e)} = \sum_j (\Delta_s - i\frac{\gamma_{se}+\gamma_{sg}}{2}) |s_j\rangle\langle s_j| + \sum_j (\Delta_r - i\frac{\gamma_{re}+\gamma_{rg}}{2}) |r_j\rangle\langle r_j|$ is the inverse of the non-Hermitian Hamiltonian of the quantum jump formalism. We define $\gamma_r = \gamma_{re} + \gamma_{rg}$ and $\gamma_s = \gamma_{se} + \gamma_{sg}$, $\tilde{\Delta}_r = \Delta_r - i\frac{\gamma_r}{2}$ and $\tilde{\Delta}_s = \Delta_s - i\frac{\gamma_s}{2}$ for more simply formula below. Substituting Eq. (4) into Eq. (6), we obtain

$$\begin{aligned}
\hat{H}_{\text{eff}} = &- \sum_j \left(\frac{|g_{r,j}|^2}{\tilde{\Delta}_r} |g_j\rangle\langle g_j| \hat{c}^\dagger \hat{c} + \frac{|g_{s,j}|^2}{\tilde{\Delta}_s} |e_j\rangle\langle e_j| \hat{c}^\dagger \hat{c} \right) \\
&- \sum_j \left(\frac{|\Omega_s|^2}{4\tilde{\Delta}_s} |g_j\rangle\langle g_j| + \frac{|\Omega_r|^2}{4\tilde{\Delta}_r} |e_j\rangle\langle e_j| \right) \\
&+ \sum_j (\lambda_{r,j} \hat{c}^\dagger |g_j\rangle\langle e_j| + \lambda_{r,j}^* |e_j\rangle\langle g_j| \hat{c}) \\
&+ \sum_j (\lambda_{s,j}^* |g_j\rangle\langle e_j| \hat{c} + \lambda_{s,j} \hat{c}^\dagger |e_j\rangle\langle g_j|) \ ,
\end{aligned} \tag{7}$$

with $\lambda_{r,j} = -\frac{\Omega_r^* g_{r,j}}{2\tilde{\Delta}_r}$ and $\lambda_{s,j} = -\frac{\Omega_s^* g_{s,j}}{2\tilde{\Delta}_s}$. To a good approximation, we can assume that $g_{r,j} = g_r$ and $g_{s,j} = g_s$. The is reasonable when the waist of a running wave cavity mode is much larger than the transversal dimension of the spin sample. In the dispersive regime, we have $|\Delta_r| \gg \gamma_r, g_r, \Omega_r$ and $|\Delta_s| \gg \gamma_s, g_s, \Omega_s$ that $\Delta_r \approx \tilde{\Delta}_r$ and $\Delta_s \approx \tilde{\Delta}_s$. We set

$$\begin{aligned} \frac{|g_r|^2}{\tilde{\Delta}_r} =& \frac{|g_s|^2}{\tilde{\Delta}_s} , \\ \lambda_{r,j} =& \lambda_{s,j} = \lambda , \end{aligned} \tag{8}$$

requiring the conditions $\frac{|d_{rg}|^2}{\Delta_r} = \frac{|d_{se}|^2}{\Delta_s}$ and $\frac{\Omega_r}{\Omega_s} = \frac{d_{rg}}{d_{se}}$ for assumption that all are real numbers. Here d_{rg} and d_{se} are the dipole moment of atomic transitions of $|r\rangle \leftrightarrow |g\rangle$ and $|s\rangle \leftrightarrow |e\rangle$. In the ground-state subspace, we can treat the atoms as a spin-1/2 system. We define the collective operators for the spins, $J_z = \sum_j (|e_j\rangle\langle e_j| - |g_j\rangle\langle g_j|)/2$, $J_+ = J_-^\dagger = \sum_j |e_j\rangle\langle g_j|$ and $\bar{J}_x = (J_+ + J_-)/2\sqrt{N_a}$. Neglecting the constant energy, the energy of the cavity and spins become $\omega_c = \delta_{\text{cav}} - \frac{1}{2} N_a \left(\frac{|g_r|^2}{\Delta_r} + \frac{|g_s|^2}{\Delta_s} \right)$ and $\omega_q = \frac{|\Omega_s|^2}{4\Delta_s} - \frac{|\Omega_r|^2}{4\Delta_r}$. Combining the energy of the cavity, we can write the Dicke Hamiltonian as

$$H_{\text{Dicke}} = \omega_c \hat{c}^\dagger \hat{c} + \omega_q J_z + 2\sqrt{N_a} \lambda (\hat{c}^\dagger + \hat{c}) \bar{J}_x . \tag{9}$$

Note that ω_q is vanishing small under the condition Eq. (8).

Lindblad superoperators for dissipation

Here we derive the Lindblad superoperators for modeling the dissipation of the spins in the master equation.

The effective Lindblad operators in Eq. (5) is [1]

$$\hat{L}_{\text{eff}}^{(k,j)} = \sum_j \hat{L}^{(k,j)} \left[\hat{\tilde{H}}_{\text{NH}}^{(j)} \right]^{-1} \hat{V}_+ , \tag{10}$$

with $\hat{\tilde{H}}_{\text{NH}}^{(j)} = (\Delta_s - i\frac{\gamma_{se}+\gamma_{sg}}{2})|s_j\rangle\langle s_j| + (\Delta_r - i\frac{\gamma_{re}+\gamma_{rg}}{2})|r_j\rangle\langle r_j|$. Substituting Eq. (4) into Eq. (10), the final form for an ensemble of individual atoms reads

$$\sum_{k,j} \mathscr{L}_{\text{eff}}(\hat{L}_{(\text{eff})}^{(k,j)}) \rho , \tag{11}$$

with

$$\begin{aligned}
\hat{L}_{\text{eff}}^{(rg,j)} &= \sqrt{\gamma_{rg}}\frac{g_{r,j}}{\Delta_r}\hat{c}|g_j\rangle\langle g_j| + \sqrt{\gamma_{rg}}\frac{\Omega_r}{2\Delta_r}|g_j\rangle\langle e_j| \,, \\
\hat{L}_{\text{eff}}^{(re,j)} &= \sqrt{\gamma_{re}}\frac{g_{r,j}}{\Delta_r}\hat{c}|e_j\rangle\langle g_j| + \sqrt{\gamma_{re}}\frac{\Omega_r}{2\Delta_r}|e_j\rangle\langle e_j| \,, \\
\hat{L}_{\text{eff}}^{(sg,j)} &= \sqrt{\gamma_{sg}}\frac{g_{s,j}}{\Delta_s}\hat{c}|g_j\rangle\langle e_j| + \sqrt{\gamma_{sg}}\frac{\Omega_s}{2\Delta_s}|g_j\rangle\langle g_j| \,, \\
\hat{L}_{\text{eff}}^{(se,j)} &= \sqrt{\gamma_{se}}\frac{g_{s,j}}{\Delta_s}\hat{c}|e_j\rangle\langle e_j| + \sqrt{\gamma_{se}}\frac{\Omega_s}{2\Delta_s}|e_j\rangle\langle g_j| \,.
\end{aligned} \tag{12}$$

It is noticeable that this collective dissipation induced by the decay channels from the excited states into the ground-state subspace is suppressed by $|g_{k,j}|^2/\Delta_k^2$ or $|\Omega_k|^2/\Delta_k^2$ with $k = r, s$. In our investigation, $|g_{k,j}|^2/\Delta_k^2 < 10^{-5}$, $|\Omega_k|^2/\Delta_k^2 < 10^{-3}$ and $\gamma_k/2\pi < 4$ MHz ($k \in \{rg, re, sg, se\}$). Thus, we can neglect the decoherence resulted from the decay of excited states in the dispersive regime [2–4].

Pure dephasing

If the spins are an ensemble of SiV centers in diamond, the ground state suffers the pure dephasing with a rate Γ_ϕ [5]

$$\mathscr{L}_\phi\rho = \sum_j \mathscr{L}(\sqrt{\frac{\Gamma_\phi}{4}}(|e_j\rangle\langle e_j| - |g_j\rangle\langle g_j|))\rho \,. \tag{13}$$

Substituting the collective operators $\sum_j(|e_j\rangle\langle e_j| - |g_j\rangle\langle g_j|)/2 = J_z = \hat{a}^\dagger\hat{a} - \mathscr{N}/2$ to this equation, we have the pure dephasing in the Bosonic picture

$$\mathscr{L}_\phi\rho = \mathscr{L}(\sqrt{\frac{\Gamma_\phi}{2}}(\hat{a}^\dagger\hat{a} - \mathscr{N}/2))\rho \,. \tag{14}$$

Lindblad superoperators for the total decoherence

According to the above analysis, the total decoherence of the system dominantly attributes to the dissipation of the cavity and the pure dephasing. It can be modeled using the Lindblad superoperators in the HP picture as

$$\mathscr{L}\rho = \mathscr{L}_c(\sqrt{\kappa}\hat{c})\rho + \mathscr{L}(\sqrt{\Gamma_\phi/2}J_z)\rho \,. \tag{15}$$

---

* keyu.xia@nju.edu.cn

[1] Florentin Reiter and Anders S. Sørensen, "Effective operator formalism for open quantum systems," Phys. Rev. A **85**, 032111 (2012).

[2] Lewis A. Williamson, Yu-Hui Chen, and Jevon J. Longdell, "Magneto-optic modulator with unit quantum efficiency," Phys. Rev. Lett. **113**, 203601 (2014).

[3] Christopher O'Brien, Nikolai Lauk, Susanne Blum, Giovanna Morigi, and Michael Fleischhauer, "Interfacing superconducting qubits and telecom photons via a rare-earth-doped crystal," Phys. Rev. Lett. **113**, 063603 (2014).

[4] Keyu Xia and Jason Twamley, "Solid-state optical interconnect between distant superconducting quantum chips," Phys. Rev. A **91**, 042307 (2015).

[5] Keyu Xia and Jörg Evers, "Ground state cooling of a nanomechanical resonator in the nonresolved regime via quantum interference," Phys. Rev. Lett. **103**, 227203 (2009).
